# Common Problems That Impact Peer Review

**DOI:** 10.1093/geroni/igab046.760

**Published:** 2021-12-17

**Authors:** Rozalyn Anderson

**Affiliations:** University of Wisconsin-Madison, Madison, Wisconsin, United States

## Abstract

This presentation will review the most common issues that affect how reviewers see a manuscript submission. These include clarity, use of figures, and attention to existing research, especially establishing the significance and novelty of the work, and how to frame a narrative. I will also address responding to peer review. The focus will be on the biological science perspective (Journals of Gerontology Series A), but these issues are relevant to all submissions to GSA journals.

